# Cellulose-*g*-poly(2-(dimethylamino)ethylmethacrylate) Hydrogels: Synthesis, Characterization, Antibacterial Testing and Polymer Electrolyte Application

**DOI:** 10.3390/gels8100636

**Published:** 2022-10-07

**Authors:** Roko Blažic, Dajana Kučić Grgić, Marijana Kraljić Roković, Elvira Vidović

**Affiliations:** Faculty of Chemical Engineering and Technology, University of Zagreb, Marulićev trg 19, 10000 Zagreb, Croatia

**Keywords:** antimicrobial activity, 2-(dimethylamino)ethyl methacrylate, gamma irradiation, hydrogel, hydrogel electrolytes

## Abstract

Hydrogels have been investigated due to their unique properties. These include high water content and biocompatibility. Here, hydrogels with different ratios of poly(2-(dimethylamino)ethylmethacrylate) (PDMAEMA) were grafted onto cellulose (Cel-*g*-PDMAEMA) by the free radical polymerization method and gamma-ray radiation was applied in order to increase crosslinking and content of PDMAEMA. Gamma irradiation enabled an increase of PDMAEMA content in hydrogels in case of higher ratio of 2-(dimethylamino)ethyl methacrylate in the initial reaction mixture. The swelling of synthesized hydrogels was monitored in dependence of pH (3, 5.5 and 10) during up to 60 days. The swelling increased from 270% to 900%. Testing of antimicrobial activity of selected hydrogel films showed weak inhibitory activity against *Escherichia coli*, *Pseudomonas aeruginosa*, and *Bacillus subtilis*. The results obtained by the cyclic voltammetry (CV) and electrochemical impedance spectroscopy (EIS) indicate that chemically synthesized hydrogels have good characteristics for the supercapacitor application.

## 1. Introduction

Hydrogels are three-dimensional polymer networks of natural or synthetic materials capable of adsorbing and retaining significant amounts of water without being dissolved [1]. A number of naturally occurring materials exhibit a hydrogel structure. The fact that the extracellular matrix (ECM) hydrogels are water-swollen fibrillary three-dimensional (3D) networks speaks of the importance and prevalence of such a structure. Naturally-derived hydrogels can be classified into three groups: protein-based materials, polysaccharide-based materials and those derived from decellularized tissue [2]. These types of hydrogels include ones derived from collagen, gelatin, elastin, fibrin and silk fibroin. Elastin and fibrin are widely found proteins in the ECM structure, and collagen is the major component inside ECM. They give ECM the required strength and elasticity to function properly, making them very promising materials for tissue engineering and cell culture systems [2,3]. The first applications of synthetic hydrogels after their synthesis were for medical reasons such as optical lenses [4,5]. They were later used for wound dressings [6], implantable medical devices, artificial blood vessels and heart valves [2,3], matrices for bone and cartilage tissue engineering [7,8] as well as drug delivery systems [9,10,11]. Due to their properties, they were also used widely in pharmacy [12,13,14], agriculture [15,16,17] and water retention [18,19]. Hydrogels are able to retain a large amount of water or biological fluids under physiological conditions and are characterized by a high degree of flexibility. Their consistency is similar to living tissues making them an ideal substance for a variety of applications. The characteristic properties of hydrogels, such as desired functionality, biocompatibility and in some cases reversibility or sterilizability meet important requirements to treat or replace tissues and organs, or interact safely with the biological system [20,21]. Recently, their application for water treatments, i.e., as adsorbent for removing heavy metal ions from wastewater has been particularly intensively researched [22,23,24,25]. In addition, the preparation of hydrogels with antibacterial properties is often achieved by incorporating silver particles, which are known to have good antimicrobial properties. In the preparation of these hydrogel–silver particle composites, the free spaces between the polymer molecules in the hydrogels act as nanoreactors, allowing control of the size and shape of the silver nanoparticles [26,27].

Smart hydrogels or stimulus-responsive hydrogels, which can display, for example, antibacterial properties, have dramatic volume changes in response to external environments, such as temperature, pH and certain stimuli, and are considered a special subgroup of materials attracting research attention. The choice of materials for stimuli is limited. One of the commonly chosen stimuli is poly(*N*-isopropylmethacrylamide [28] or poly(2-(dimethylamino)ethylmethacrylate) (PDMAEMA). They are pH and temperature double-responsive [29] and possess antibacterial activity [30,31]. More than two decades ago, PDMAEMA and its copolymers were synthesized and evaluated as gene transfer agents and carrier systems for DNA [32,33]. Often, they are combined with petrochemical based polymers [34] or biobased polymers [35,36]. Water swelling tests on interpenetrating network (IPN) hydrogels based on nanofibrillated cellulose (NFC) and PDMAEMA prepared via crosslinking free radical polymerization showed that the IPN hydrogels were both pH-sensitive and temperature-sensitive. The swelling of hydrogels was limited at high temperature and in neutral medium. There was an increase in the swelling ratio as the NFC content increased. The synthesized materials were tested for removal of Pb(II) and Cu(II) ions. The achieved adsorption of both ions was better in comparison with other literature data (more than 50% for Cu, and several times higher for Pb). The high porosity and uniform pore structure, among other characteristics, presumably contributed to removal of Pb(II) and Cu(II) ions [25].

In this study, we aimed at preparing hydrogels from PDMAEMA grafted onto cellulose (Cel-*g*-PDMAEMA) by a free radical polymerization method, chemical synthesis, as well as by applying gamma irradiation in an attempt to increase crosslinking and content of PDMAEMA in hydrogels. Regarding the possible application of hydrogels the antimicrobial activities of selected films that are additionally decorated with silver were also tested. The cyclic voltammetry (CV) and electrochemical impedance spectroscopy (EIS) were used to test potential application of chemically synthesized hydrogels as sheets in the assembled supercapacitor.

## 2. Results and Discussion

### 2.1. Polymerization Reaction

**Figure 1:**
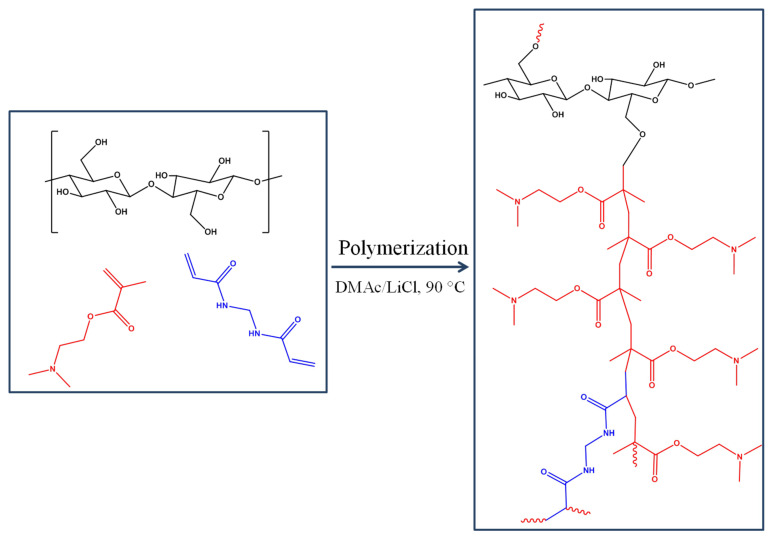
The chemical reaction of polymerization and grafting of poly(2-(dimethylamino)ethylmethacrylate) (PDMAEMA) onto cellulose to produce networks Cel-*g*-PDMAEMA crosslinked with MBA.

The polymerization of 2-(dimethylamino)ethyl methacrylate (DMAEMA) with *N*,*N*-methylenebis (acrylamide) (MBA) in a cellulose solution is a complex process that can lead to different products [37]: (a) polymer grafted onto cellulose, (b) a semi-interpenetrating network formed by crosslinking the polymer, (c) homopolymer or copolymer (branched structure), and (d) polymer microgel. A detailed study of the structure of the prepared Cel-*g*-PDMAEMA hydrogels with different molar ratios of cellulose to DMAEMA (1:1, 1:3, and 1:5), especially with respect to the content of PDMAEMA microgel, was undertaken in other research [38]. The chemical reactions of grafting PDMAEMA onto cellulose and crosslinking with MBA to form a network with(out) gamma irradiation are shown in Figure 1.

**Scheme 1:**
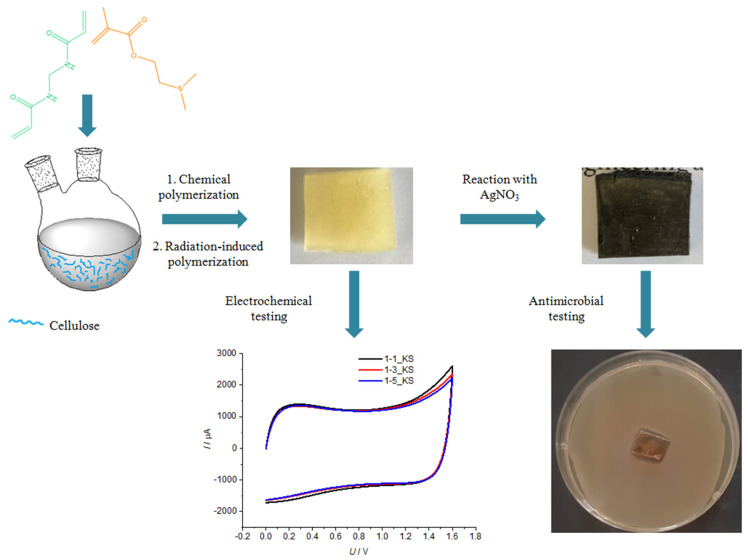
Methodology for Cel-*g*-PDMAEMA hydrogel preparation and testing regarding potential applications.

In this work, the focus is on the swelling of Cel-*g*-PDMAEMA hydrogels over a broader pH range. In addition, the effect of PDMAEMA on the antibacterial properties of copolymer hydrogels as well as on the efficiency of hydrogel decoration with silver was investigated, where improvement is expected due to the presence of nitrogen atoms. The analysis of the electrochemical properties of the selected hydrogels indicates their suitability for application as supercapacitors (Scheme 1). Therefore, the obtained results indicate a possible application of the synthesized materials as separators, for example, in electrochemical devices (capacitors, etc.) or in the medical field (dressings, wraps, polymers for controlled delivery of drugs, etc.).

Cellulose grafted with PDMAEMA and crosslinked with MBA hydrogels are named Cel-*g*-PDMAEMA. Samples are referred as series, generally x-y_KS or x-y_Z where x-y represents the ratio *n*(cellulose)/*n*(DMAEMA):1-1, 1-3 or 1-5, KS represents hydrogels prepared via chemical synthesis, while Z (10, 30 or 100) represents the irradiation dose in kGy.

### 2.2. Structural Characterization of Synthesized Cel-g-PDMAEMA Hydrogels

#### 2.2.1. FTIR Spectroscopy Analysis

**Figure 2:**
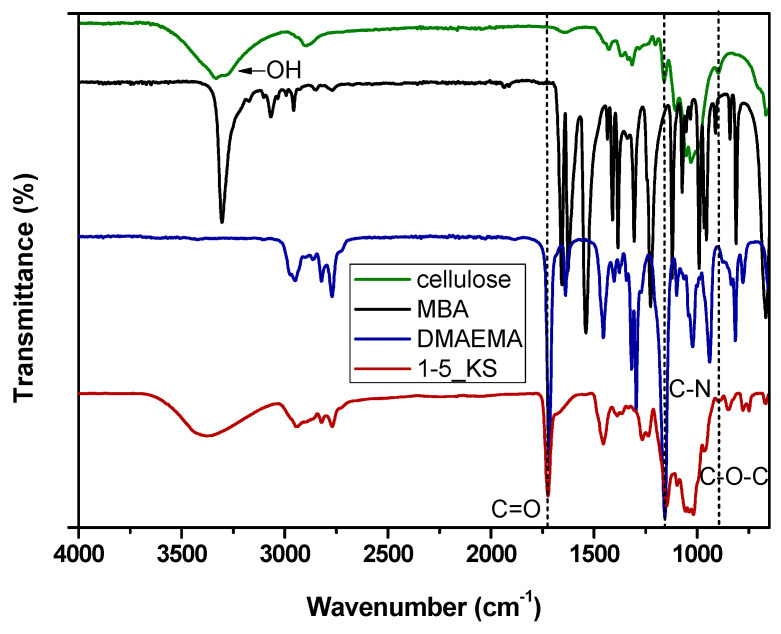
FTIR spectra of microcellulose, *N*,*N*-methylenebis (acrylamide) (MBA), 2-(dimethylamino)ethyl methacrylate (DMAEMA) and Cel-*g*-PDMAEMA network crosslinked with MBA sample synthesized chemically.

The FTIR spectrum of microcellulose is shown in Figure 2. In the wavenumber range between 3000 and 3600 cm^−1^, there is a broad signal with a maximum at 3331 cm^−1^, which is characteristic of the vibrational band of the hydroxyl group in the polysaccharide, which is affected by intra- and intermolecular hydrogen bonding.

The band at 2896 cm^−1^ belongs to the vibrations of the CH bonds. The band at 1634 cm^−1^ corresponds to the vibrations of the water molecules absorbed in the cellulose. The bands at 1428 and 1030 cm^−1^ are characteristic of the cellulose and belong to the vibrations of the groups CH_2_ and OH in the cellulose. The band at 897 cm^−1^ corresponds to the vibration of the characteristic *β*-(1 → 4) glycosidic bond [39,40]. 

The FTIR spectrum of MBA presented in Figure 2 shows a band with maxima at 3304 cm^−1^ assigned to N-H stretching vibrations. Bands corresponding to the stretching vibrations of CH_2_ appear at 3067 and 2956 cm^−1^. The strong band at 1656 cm^−1^ is assigned to the C=O stretching mode (Amide I band), while the strong band at 1535 cm^−1^ is assigned to the N-H deformation (Amide II band). The strong band around 1620 cm^−1^ indicates the presence of a C=C double bond and its stretching mode. Moreover, the medium–strong band at 1428 cm^−1^ is assigned to the in-plane scissoring or bending of CH_2_. The strong band around 1380 cm^−1^ is assigned to the out-of-plane bending of CH_2_, while the band at 987 cm^−1^ is assigned to the vibration of CH_2_, respectively [41,42].

Figure 2 shows the FTIR spectrum of DMAEMA with the maxima at 2949 cm^−1^ corresponding to the sp^3^ vibration of the CH bond. The signals at 2822 and 2770 cm^−1^ also correspond to sp^3^ vibrations of the CH bond, but within the N(CH_3_)_2_ group. The signal at 1717 cm^−1^ corresponds to the vibration of the carbonyl group, while at 1638 cm^−1^ the signal belonging to the vibration of the C=C bond is visible. In addition, an adsorption band appeared at 1452 cm^−1^, which is characteristic of the bending of CH_2_. The adsorption band with a maximum at 1150 cm^−1^ results from the C-N stretching vibrations [39,43].

The infrared spectrum of the 1-5_KS sample with the highest amount of DMAEMA in the reaction mixture is also shown in Figure 2. In this hydrogel apart from a broad peak around 3330 cm^−1^, which is characteristic for cellulose, the signals characteristic for PDMAEMA appear at 2940, 2828 and 2770 cm^−1^. There was a band from the stretching vibrations of the C=O bond, with a maximum at 1724 cm^−1^, which was shifted to higher wavenumbers by 7 units compared to the position of the same band in the FTIR of the DMAEMA monomer (Figure 2). Additionally, the band from stretching vibrations of the C-N and C-O groups with a maximum at 1148 cm^−1^ was shifted by 8 units to lower wavenumbers compare to the position in the DMAEMA. The band at 1455 cm^−1^ from CH_2_ bending occurred at the same wavenumber. In the 1-5_KS hydrogel spectra, there were no bands from the double C=C bond and from the vinyl group, indicating that the bonding of the DMAEMA monomers and crosslinking with the MBA occurs by breaking the C=C bonds. 

**Figure 3:**
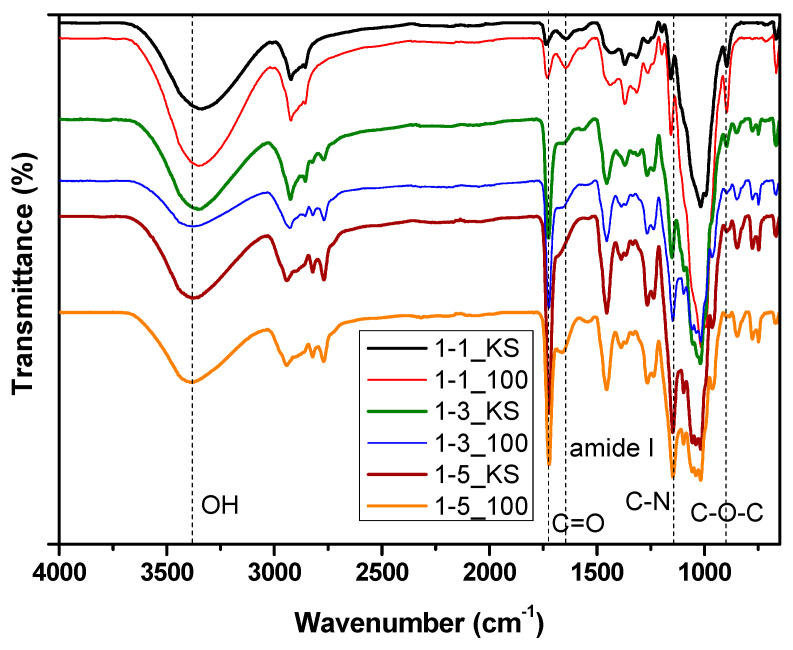
FTIR spectra of Cel-*g*-PDMAEMA hydrogel films (cellulose:DMAEMA = 1:1, 1:3, 1:5) prepared after chemical reaction (KS) and additionally irradiated with 100 kGy.

Figure 3 shows FTIR spectra of hydrogel films prepared from synthesized Cel-*g*-PDMAEMA after chemical reaction and additionally irradiated with 100 kGy. The band characteristic of the stretching vibrations of the C=O bond was strong and appeared at the same wavenumber, 1724 cm^−1^, in samples 1-3 and 1-5. In samples 1-1, the C=O signal was weak and shifted to 1731 and 1737 cm^−1^, respectively, while the band of amide band I appeared with similar intensity at 1648 cm^−1^. Intramolecular hydrogen bonds could be formed via the C=O group of PDMAEMA and the N–H group of MBA. The shift of these maxima to lower wavenumbers suggests that the N–H and C=O groups of PDMAEMA and MBA are involved in the hydrogen bond formation. The results of the FTIR analysis are in agreement with the literature data [40,41,42,43,44].

By comparing the intensity ratio of the band characteristic of PDMAEMA (C=O) and the band characteristic of the cellulose (*β*-(1 → 4) glycosidic bond occurring at 897 cm^−1^), it was possible to follow the increase in PDMAEMA content in the hydrogel in agreement with its increased content in the reaction mixture (Table 1). 

The ratio *I*_1725_/*I*_895_ increased from 0.5 in 1-1_KS up to 4.5 and 13.3 in 1-3_KS and 1-5_KS samples. The absorption of irradiation caused further increase of PDMAEMA in hydrogels of series 1-3_100 and 1-5_100 but did not influence sample 1-1_100, as its ratio *I*_1725_/*I*_895_ reveals [45]. The primary radiation damage is the formation of carbon-centered radicals. In the case of cellulose, five different types of radicals produced by H atom elimination from a C-H bond are expected and all the final radiation chemical changes of cellulose are consequences of unimolecular or bimolecular reactions of the radicals. Subsequently, *β* cleavage of the radical can lead to the breaking of the glucoside bond or opening of the anhydroglucose ring. In both cases carbonyl groups are produced as a result of the cleavage. In case of series 1-1 there is no change in the ratio *I*_1725_/*I*_895_ (Table 1), which would imply that neither radiation damaged cellulose nor enhanced incorporation of PDMAEMA into the hydrogel. The applied dose at 100 kGy does not cause significant damage to cellulose, which is in accordance with the literature where it is mostly claimed that doses of more than 100 kGy cause destruction [46,47]. Irradiation did not increase the content of PDMAEMA, but its presence, even small, affects the structure, porosity and swelling ability of the material, as will be seen below.

It was mentioned earlier that these kinds of reactions, which include chemical synthesis and irradiation, are complex processes that can result in different products: (a) polymer grafted onto cellulose, (b) a semi-interpenetrating network formed through crosslinking of the polymer, (c) homopolymer or copolymer (branched structure), and (d) polymer microgel. Here, as well as during the final design of spheres or films that includes freeze-extraction, numerous influences intertwine that causes phase separation, chain orientation, and different crosslinking densities are possible, and all of this leads to differences in the composition and structure of the material. A detailed study of the influence of an irradiation dose on the structure of Cel-*g*-PDMAEMA hydrogels was conducted [38,43].

Furthermore, in the spectra in Figure 3, one can see that with the increasing ratio of PDMAEMA, the signals characteristic of the N(CH_3_)_2_ group at 2940, 2828 and 2770 cm^−1^ become pronounced. Samples with a higher ratio of PDMAEMA display a strong signal at 1457 cm^−1^ characteristic for bending of CH_2_ in PDMAEMA. The intensity of the band from stretching vibrations of the CO group, *ν*_s_(C–O), with a maximum at 1157 cm^−1^ relative to the intensity of *β*-glycosidic linkages (897 cm^−1^) in sample 1-1 and cellulose, are similar. With an increasing amount of PDMAEMA in samples 1-3 and 1-5 the relative increase of the signal at 1157 cm^−1^ against the two signals characteristic of cellulose (897 and 1020 cm^−1^) was significant. It was more conspicuous in the case of irradiated hydrogels. In addition, numerous small signals confirm the presence of methacrylate compounds in the hydrogel. Weakly separated signals at 1235 cm^−1^ and 1265 cm^−1^, i.e., “shoulder” at 1296 cm^−1^, originate from the presence of MBA (1226 cm^−1^) and PDMAEMA (1296 cm^−1^), respectively. Additionally, signals recorded in the fingerprint region (851 cm^−1^ and 780 cm^−1^) originate from MBA and PDMAEMA (Figure 3).

In hydrogels 1-1_KS and 1-1_100 instead of a strong band from the stretching vibrations of the C=O bond, *ν*(C=O) with a maximum at 1724 cm^−1^ a weak band was recorded. Simultaneously, a relatively stronger intensity amide band I appeared at 1648 cm^−1^. Intramolecular hydrogen bonds could be formed via the C=O group from PDMAEMA and N–H group from MBA. The shifting of these maxima towards lower wavenumbers indicated that the N–H and C=O groups of PDMAEMA and MBA participated in the hydrogen bond formation. Additionally, intensity of the band from stretching vibrations of the CO group, *ν*_s_(C–O), with a maximum at 1157 cm^−1^, displayed an intensity ratio to the *β*-glycosidic linkages similar to that in pure cellulose (897 cm^−1^). With increasing amount of PDMAEMA these signals both displayed a significant increase relative to the band at 897 cm^−1^, whereat it was more conspicuous in the case of irradiated hydrogels.

#### 2.2.2. Scanning Electron Microscopy Analysis

**Figure 4:**
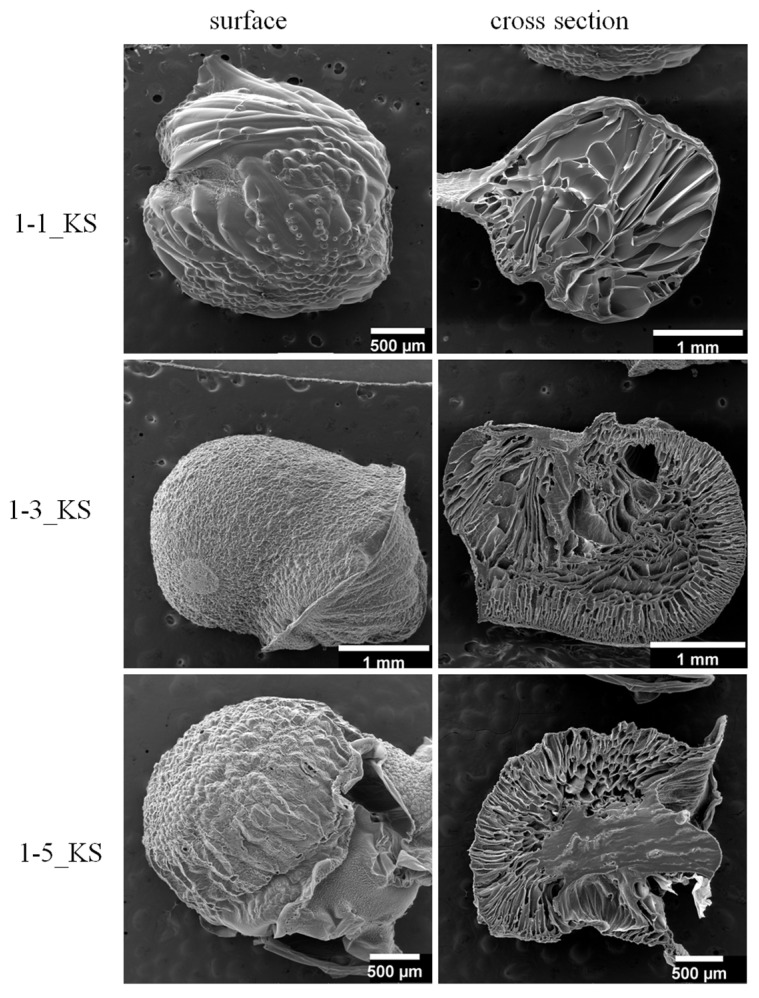
SEM micrographs of outer sphere surface and cross-section of sphere for samples 1-1_KS, 1-3_KS and 1-5_KS.

To characterize the morphologies of the Cel-*g*-PDMAEMA hydrogel sample (both spheres and films) swollen at equilibrium, the SEM micrograms were obtained. Figure 4 shows micrographs of the outer surface and cross-section of the sphere for samples 1-1_KS, 1-3_KS, and 1-5_KS. The surface of the spheres differs in roughness, although not significantly. However, the porosity pattern on the cross-section differs significantly depending on the composition. Drying material by the freeze-extraction method enables the preparation of porous materials because the pores are not crushed by capillary force, therefore all samples prepared by the freeze-extraction method have a porous structure. The size distribution of the pores changes from the outer layer (edge) of the sphere towards its center. In the outer layer, near the edge of the sphere, there are smaller pores (<50 µm), while the pores in the center of the sphere are larger. The smaller pores are the result of the formation of smaller water crystals during the cooling/freezing of the spheres in the cryostat at −40 °C. The slower cooling in the center of the sphere contributed to the formation of larger water crystals and subsequently to the formation of larger pores. The pore size and the distribution of the pores over the cross-section of the spherical samples clearly show the influence of the composition and design protocol of the hydrogels.

**Figure 5:**
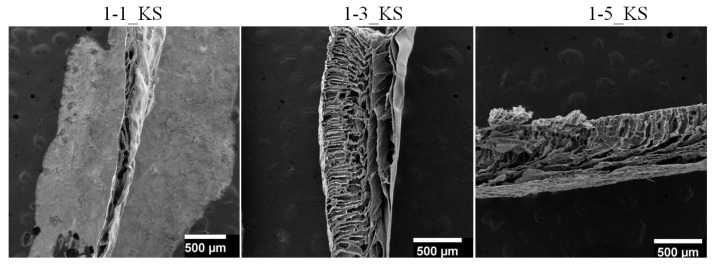
SEM micrographs of Cel-*g*-PDMAEMA hydrogel films swollen in the equilibrium state.

Figure 5 shows SEM micrographs of the Cel-*g*-PDMAEMA hydrogel samples shaped into films. Again, a difference in bulk structure (in cross-section) can be seen depending on the composition of the material. Individually, the three-dimensional structure of the hydrogels looked like a semi-uniform cross-linked network, with a more or less thin layer of different structure formed when the hydrogel films were made, as one side was in contact with the glass surface and the other side was in contact with the air. If necessary, this characteristic could be modified by applying a different film casting procedure. The thickness of the swollen films was 0.15–1 mm. The cross-section shows differences between hydrogels depending on their composition. Hydrogel 1-1_KS has a fairly uniform, dense structure except for the thin porous edge layer. Hydrogel 1-3_KS has a very porous cross-section in which two perpendicular layers with different pore sizes can be seen. A similar structure is present in hydrogel 1-5_KS, which is characterized by high porosity. Here, the difference between the porous layers is less pronounced. Similar to the spheres, this structural organization especially in 1-3_KS and 1-5_KS hydrogels provided a lot of free space within the cross-linked polymer network. Therefore, they appear to be suitable for various applications where fluid sorption within the network is required, including carriers for many active substances.

Comparing the pore size in samples with different geometries (spheres and films), it can be seen that for spheres 1-1_KS, 1-3_KS and 1-5_KS, the pore size in the swollen state was initially in the range of 40–455 µm, 60–355 µm and 60–180 µm, respectively (Figure 4). The pore size in the layer near the sphere surface was <60 µm for samples 1-3_KS and 1-5_KS. Detailed pore size distribution for initial spheres is presented in Figure S1. At the same time, the pore size of the prepared film samples in the swollen state was in the range of 25–120 µm, 15–185 µm and 15–140 µm, respectively (Figure 5). Detailed pore size distribution for prepared films is presented in Figure S2. Based on the average pore size, the synthesized materials can be classified as macroporous hydrogel [42,48].

**Figure 6:**
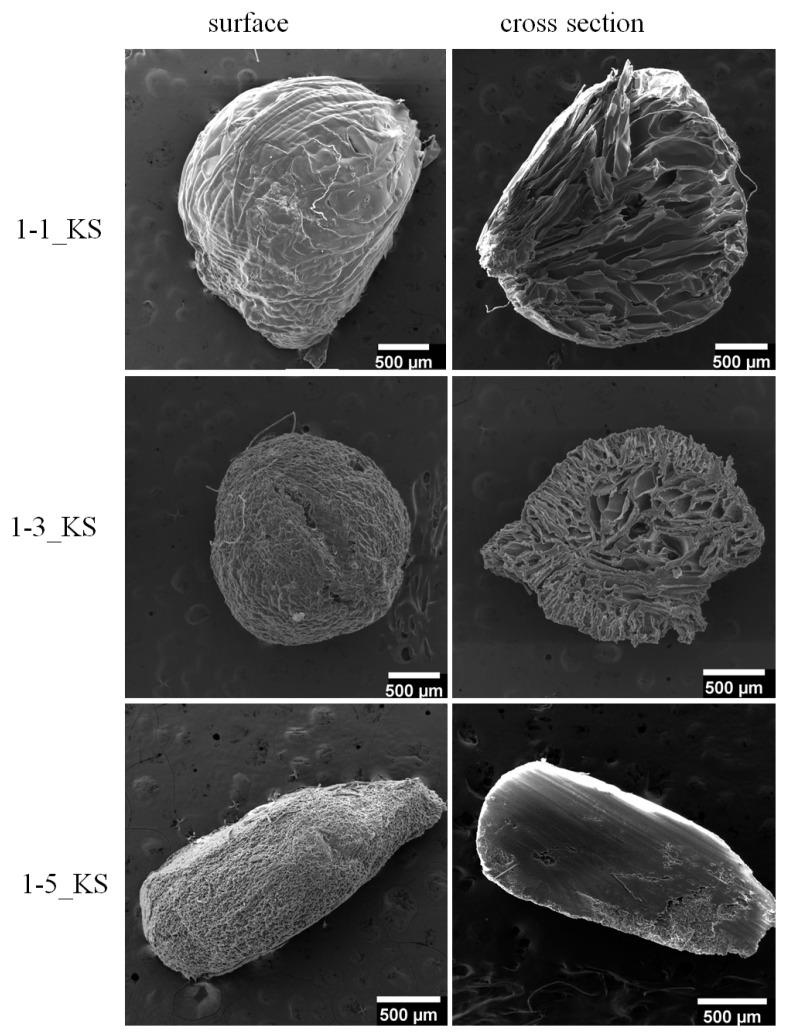
SEM micrographs of outer sphere surface and cross-section of sphere for samples 1-1_KS, 1-3_KS and 1-5_KS after 60 days of swelling in deionized water.

Figure 6 shows micrographs of the outer surface of the sphere and the cross-section of the sphere for samples 1-1_KS, 1-3_KS, and 1-5_KS after 60 days of swelling in deionized water. The spheres of 1-1_KS and 1-3_KS exhibited similar porosity accompanied by some shrinkage, while spheres 1-5_KS changed geometry from a spherical to an elliptical shape and porosity disappeared. Their surfaces became somewhat smoother, while the cross-section showed a large change from porous to dense form. This indicates that their behavior and persistence during swelling depend on composition. 

The pore size of samples 1-1_KS and 1-3_KS, which were swollen for 60 days, were in the range of 30–315 µm and 60–270 µm, respectively (Figure 6). Comparing the pores in the spheres at the beginning and after 60 days of swelling, it can be seen that their size, shape and distribution changed slightly in the samples with the lower PDMAEMA ratio (1-1_KS and 1-3_KS) (Figure S3). The spheres of the 1-5_KS samples, however, showed a complete change in structure. This can probably be correlated with the method of incorporation of PDMAEMA in high concentrations into the hydrogel and the change in its content during long-term swelling.

**Figure 7:**
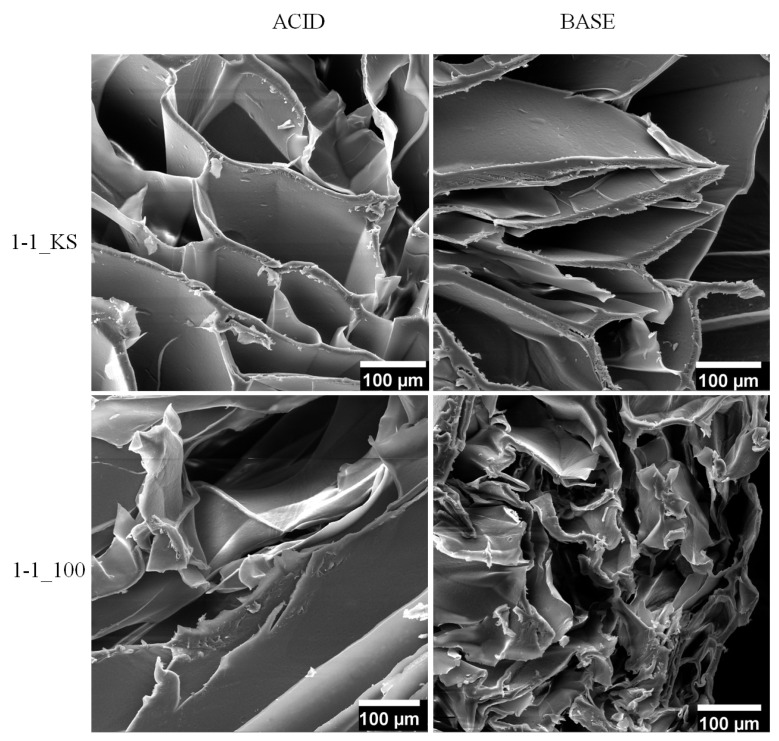
SEM micrographs of Cel-*g*-PDMAEMA hydrogel samples, swollen in the equilibrium state for sample 1-1_KS and 1-1_100.

**Figure 8:**
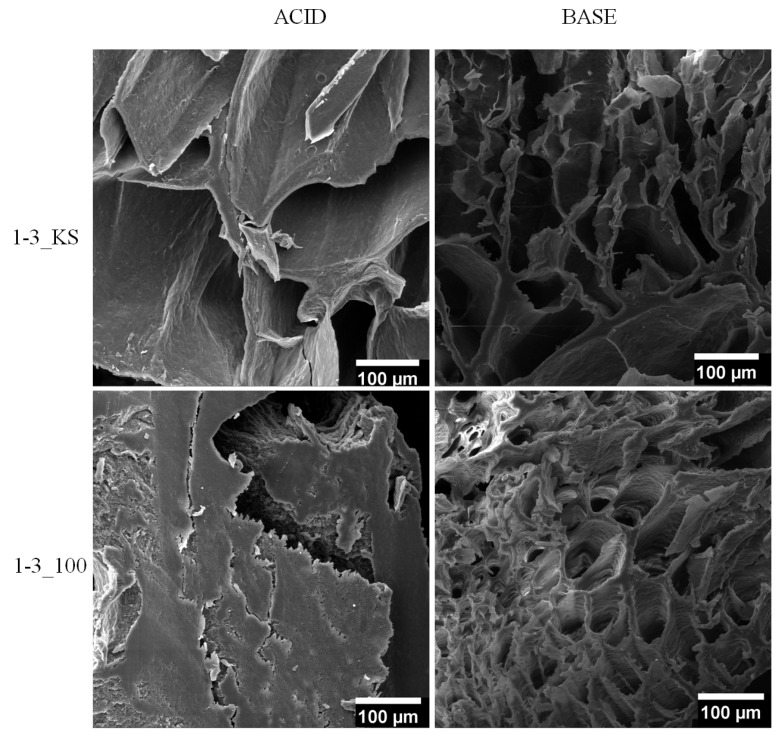
SEM micrographs of Cel-*g*-PDMAEMA hydrogel samples, swollen in the equilibrium state for sample 1-3_KS and 1-3_100.

Samples 1-1_KS and 1-1_100 as well as 1-3_KS and 1-3_100 after swelling in acidic or alkaline media for 60 days were scanned and SEM micrographs are shown in Figure 7 and Figure 8. They displayed small, up to significant, structural differences. All observed samples prepared by chemical synthesis exhibited porous structure in both media. Samples 1-1_KS and 1-3_KS, which were studied in acidic media, both exhibited a similar pore shape, while in alkaline media they had a significantly different pore structure. If we keep with the 1-1 series, the structure of sample 1-1_100 swollen in alkaline environment is the most different, being crumpled, rough and without the well-defined pore geometry. The samples of series 1-3 showed more pronounced structural differences, the largest being found in sample 1-3_100 swollen in acidic media. It is characterized by a rather compact, smooth structure in which cracks are visible, but no pores are visible. The observed major differences in the structure of the hydrogels do not show a cause-and-effect relationship with their degree of swelling. In particular, the hydrogels 1-3_100, which were swollen in acidic and alkaline media, show the same equilibrium degree of swelling after 60 days, as will be commented on later (see Section 2.3.1).

### 2.3. Swelling Study

The spheres were dried by a freeze-extraction method that enabled the design of porous materials. The behavior of Cel-*g*-PDMAEMA hydrogels swollen in deionized water (pH 5.5) was monitored at 20 °C. To investigate the influence of pH, the samples were also swollen in acidic (pH = 3) and alkaline (pH = 10) media. The swelling ratio, α, was calculated according to Equation (1). 

#### 2.3.1. Equilibrium Hydrogel Swelling at 20 °C and pH 5.5

**Figure 9:**
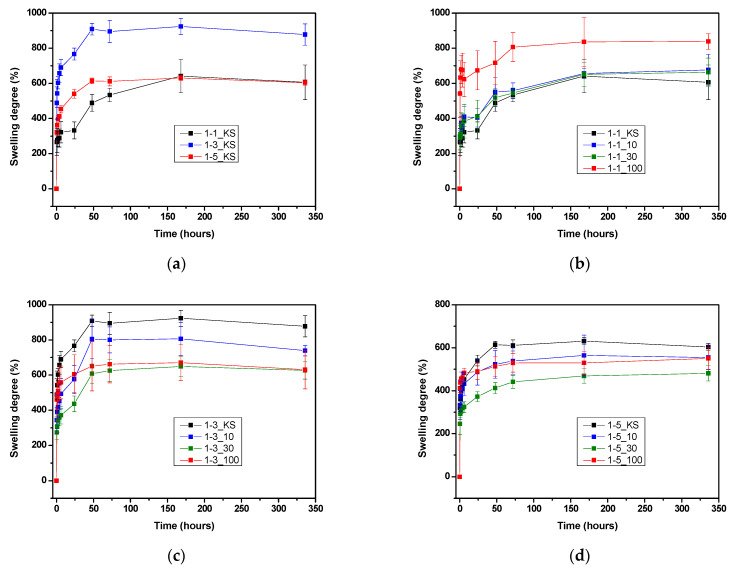
Comparison of swelling kinetic and equilibrium swelling degree for hydrogel samples with different molar ratio of cellulose and DMAEMA. (**a**) Comparison of swelling kinetic and equilibrium swelling degree for hydrogels after irradiation (**b**–**d**).

The swelling kinetics of the prepared spheres was studied in deionized water (pH 5.5) at 20 °C for 14 days, and the results are shown in Figure 9. A study of the hydrogels prepared by chemical synthesis showed their fairly extensive swelling. The more porous samples 1-5_KS and especially 1-3_KS reached the maximum degree of swelling in two days, while sample 1-1_KS, which contained predominantly larger elongated pores as Figure 4 SEM reveals, reached the maximum α in seven days. These results indicate that a higher amount of PDMAEMA affects the structure and porosity of the materials and in this way contributes to faster swelling. After reaching equilibrium samples 1-1_KS and 1-5_KS displayed similar swelling degree of ca. 640% while the highest α of ca. 925% displayed sample 1-3_KS. Perhaps the explanation of the achieved similar swelling is that the sample 1-1_KS, as already mentioned, shows mostly closed pores and the sample 1-5_KS a porous outer zone (layer) but a compact non-porous central part of the sphere. Only the sample 1-3_KS shows a very porous structure throughout the cross-section of the sphere. It is worth mentioning that sample 1-1_KS despite a negligible content of PDMAEMA (Figure 3, FTIR), when compared with the sphere of pure cellulose displayed many times higher degree of swelling. In our preliminary studies we found that the degree of swelling of the cellulose sphere was 80% after 4 h, while the sample of 1-1_KS at the same time showed a degree of swelling of ca. 300%. One can assume that the reason for the much lower swelling of the cellulose sphere is that a porous structure is not obtained despite the same preparation procedure (freeze-extraction method) as for copolymer spheres [43].

The highest degree of swelling of sample 1-1_100 among all samples in this series is not likely to be attributed either to increased ratio of PDMAEMA based on FTIR analysis or to increased cellulose degradation. Literature data related to the influence of radiation on the degradation of cellulose differ with regard to the origin and type of cellulose (microcrystalline cellulose, bacterial cellulose, pine wood cellulose, cotton-cellulose, etc.). They generally agree that the weaker radiation does not cause significant changes in the structure. Thus, it was determined that sterilization with gamma irradiation at 25 kGy caused no significant structural changes in the polymer [46] and that irradiation at 10 kGy caused decrease of molecular weight of ca. 12% [47]. On the other hand, doses of 100 kGy and above caused significant changes of cellulose properties such as molecular weight, relative crystallinity, and surface area [49].

Irradiated materials with a higher content of PDMAEMA, series 1-3 and 1-5, displayed a lower degree of swelling in comparison with hydrogel spheres prepared by chemical synthesis (Figure 9). Although there are small differences, it can be said that the swelling ability of these samples decreases with increasing irradiation dose. Due to the higher ratio of DMAEMA in the reaction mixture, and because it seems to be more pliable to irradiation compared to cellulose, its incorporation into hydrogel is enhanced, both by grafting onto cellulose or in the form of microgels. The increased proportion of PDMAEMA was confirmed by FTIR analysis. In this way, the number of possible networking sites is increased. More frequently crosslinked networks swell less. Additionally, smaller deviations from the trend are probably due to variation in porosity. Specifically, samples of series 1-3 displayed swelling degree values in the range between 630% and 925%; with the latter, the largest α was notified in the 1-3_KS after 7 days.

#### 2.3.2. Comparison Equilibrium Hydrogel Swelling at 20 °C and pH = 3.0, pH 5.5 and pH 10

**Figure 10:**
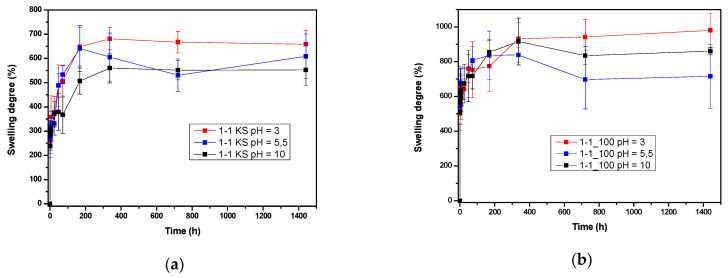

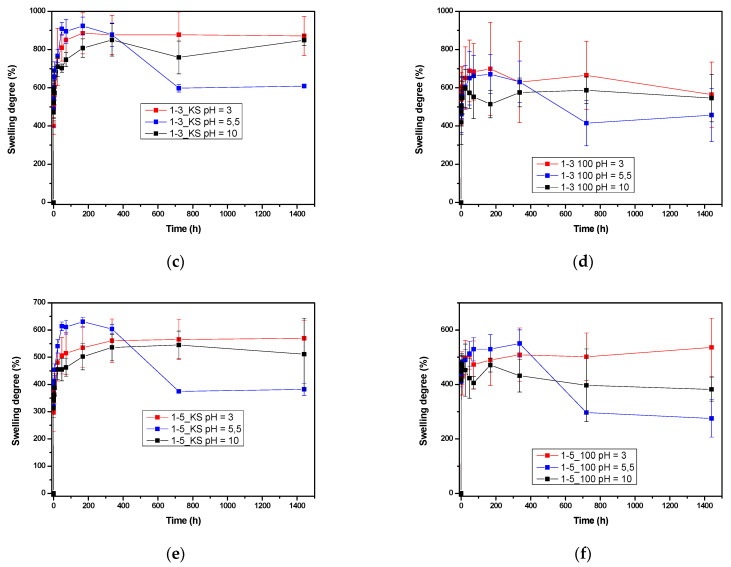
Comparison of swelling kinetic and equilibrium swelling degree for hydrogel samples at different pH for initial 1-1_KS, 1-3_KS and 1-5_KS samples (**a**,**c**,**e**) and irradiated samples 1-1_100, 1-3_100, 1-5_100 (**b**,**d**,**f**).

The influence of the pH of the medium on the swelling of Cel-*g*-PDMAEMA hydrogels is shown in Figure 10. Hydrogels prepared by chemical synthesis and those subsequently irradiated with 100 kGy are shown in parallel.

During the first week of swelling, the hydrogels mostly displayed the highest swelling ratios in deionized water (pH 5.5). In the literature, similar hydrogels, i.e., interpenetrating network (IPN) based on nanofibrillated cellulose (NFC) and PDMAEMA prepared via crosslinking free radical polymerization were tested during 24 h, at pH in a range 3 to 11 [25]. They came to the conclusion that the swelling ratios under acidic and alkaline condition are higher than those in a neutral environment, which is the opposite compared to these samples in the initial period. They explained that the phenomenon may be attributed to specific charge properties of PDMAEMA and NFC in aqueous solution. PDMAEMA is a kind of tertiary amine with a pKa of about 7.5 and NFCs have carboxyl groups on the surface with a pKa of about 4.6. Therefore, in acidic medium, the tertiary amine groups can easily be protonated with a positive charge and the polymer chains become stretched due to the electrostatic repulsion. Thus, higher swelling ratios can be achieved because of low resistance of water entering the hydrogel. In alkaline environment, they explained, carboxylic acid groups on NFC gradually shift to the carboxylate anion, which results with weaker hydrogen bonds interaction and enhanced electrostatic repulsion in hydrogels. 

Here, the hydrogels displayed higher swelling ratios under acidic and alkaline conditions only after 7 and 14 days, respectively. We can assume that it takes longer due to structural differences that slow the process and restrict stretching in spite of (de)protonation. It is only subsequently that both the repulsive structure and ionization of hydrogels occur and facilitate the diffusion of water molecules and increase the swelling ratios as Salama et al. explained [50]. Furthermore, the swelling ratio of the IPN hydrogels is also associated with the content of the NFCs. With an increase in the NFCs ratio, the swelling ratio of the hydrogel gradually increases [25]. This is also true here, with the exception of the 1-1_KS series. In general, irradiated samples with higher PDMAEMA ratio (1-3_100 and 1-5_100) display a lower degree of swelling in comparison with hydrogels prepared only by chemical reaction, which is in accordance with findings of FTIR. Unlike the latter, hydrogel 1-1_100 showed a higher α in comparison with 1-1_KS. As the previous study showed, the microgelation is very common. The distribution of microgel affects the structure through the density of the crosslinking and its subsequent migration from the loose network is facilitated. It indicates, apart from reaction mixture composition, the influence of different contributions during preparation procedure on materials properties [38]. Almost equal swelling of 1-3 samples in alkaline and acidic medium studied over a longer period makes likely its use for specific applications such as medicine, pharmaceuticals for drug delivery, sensor manufacturing, separators in electrochemical devices (batteries, capacitors and the like), etc. [44].

### 2.4. Decorating Hydrogel Films with Silver Particles

**Figure 11:**
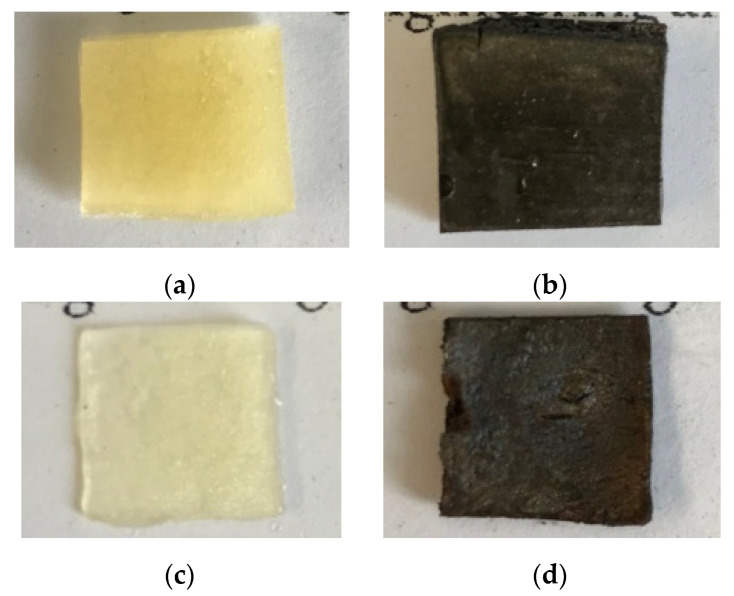
Sample 1-5_KS before (**a**) and after (**b**) immersion and sample 1-5_100 before (**c**) and after (**d**) immersion in 0.01 M AgNO_3_ during 24 h.

Synthesis of hydrogels with silver was carried out by swelling hydrogels in a silver nitrate solution. Silver atoms are formed by the reduction of silver ions from the complex, which then agglomerate and finally form stable silver particles. Silver is known for its antibacterial activity and can also be used as a catalyst. The introduction of silver into the structure of the hydrogel expands its potential applications, because by combining substances with different properties, new materials with prestigious properties can be created for very different applications, from sensors to various supports and coatings to catalysts, from pharmacy, medicine, water treatment industry and the like [51,52,53,54]. Figure 11 shows hydrogels 1-5_KS and 1-5_100 before and after incorporation of silver.

**Figure 12:**
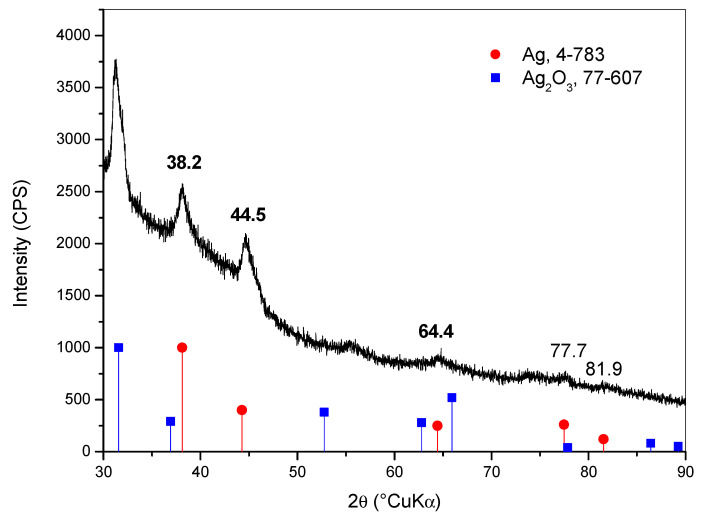
XRD diffractogram of formed silver particles in sample 1-5_100+AgNO_3_ (crystallographic database for silver (ICDD PDF No. 4-783) and Ag_2_O_3_ (ICDD PDF No. 77-607)).

It is known that the formation of silver particles in samples is accompanied by a color change, which can be considered a quick and preliminary indicator that a reduction of silver has occurred but the formation of silver particles in solution by Ag^+^ reduction is commonly and accurately monitored by UV-VIS spectroscopy. However, the formation of silver particles on solid supports, e.g., hydrogels, makes in-situ monitoring of Ag particle formation by UV-VIS spectroscopy impossible, except in rare cases when the media is transparent. At the same time, it is well known from the literature that the presence of Ag^0^ particles can be confirmed by XRD analysis both in solution and in composites [55,56]. The XRD pattern of samples 1-5_100 + AgNO_3_ is shown in Figure 12, where broad diffraction lines characteristic of silver (ICDD PDF No. 4-783) can be seen. Moreover, additional diffraction lines in the diffractogram indicate the presence of silver oxide, where the obtained pattern most closely resembles Ag_2_O_3_ (ICDD PDF No. 77-607). Due to the large surface area of nanoparticles, contact of silver nanoparticles with aqueous media can lead to oxidation and formation of silver oxide. Silver oxide also shows good antimicrobial properties as reported in the literature [57,58,59]. Gao et al. showed that silver oxide enhances antibacterial properties of material over a long period of time because the presence of silver oxide modulates the release of Ag^+^ ions [58].

### 2.5. Antimicrobial Testing

A series of samples for each Cel-*g*-PDMAEMA hydrogel: plain, silver decorated, and those both irradiated and decorated were used to study antibacterial properties against *Bacillus subtilis*, *Escherichia coli*, and *Pseudomonas aeruginosa*. The results of the antimicrobial activity of the tested samples using the disk diffusion method are summarized in Table 2 and Figures S4–S6. 

The data in Table 2 show that chemically synthesized hydrogels did not exhibit antibacterial activity, except for sample 1-1_KS, which showed a weak response against *P. aeruginosa* and *B. subtilis*. The samples of all series decorated with silver particles showed weak inhibitory antibacterial activity.

Silver nanoparticles have been proven to have antibacterial properties and due to these properties they are used in medical products as well as in consumer products such as textiles with antibacterial properties, cleaning cloths, air filters, food containers, cosmetic products, various coatings, etc. [54]. Their contribution to improving the efficacy of various antibiotics is particularly important. Various studies have shown that they change the activity of antibiotics. For example, the study of the activity of levofloxacin against *E. coli*, *B. subtilis*, *P. aeruginosa* and *S. aureus* has shown a synergistic effect [51]. Such studies are of great importance because of the worrying resistance of bacteria. They not only show a synergistic effect with antibiotics, but also reduce the dose of antibiotic used in therapy. Unlike antibiotics, silver nanoparticles act through a combination of mechanisms rather than a single one. The obtained inhibition zones indicating a stronger antimicrobial effect against all tested bacterial cultures in films decorated with AgNPs, can be explained by their physical properties, where the effect of AgNPs strongly depends on their size, shape and concentration [60]. The AgNPs displayed different activity against various bacteria. They were more active against *E. coli* and *B. subtilis* than against *P. aeruginosa* [61,62,63]. Here, the irradiated samples show moderately to several times larger zones of inhibition within a single series. This is not surprising since the use of radiation for the purpose of sterilization is well known, e.g., in medicine, food industry, etc., and it has enhanced the incorporation of PDMAEMA into the hydrogel, as determined by FTIR analysis. Studies on the antimicrobial activity of PDMAEMA have shown that it is active against both gram-positive and gram-negative bacteria [31,64]. Therefore, based on the obtained results, it can be concluded that there is a synergistic effect, i.e., higher content of PDMAEMA contributes to higher antibacterial activity, as well as the introduction of AgNPs and applied irradiation [31]. It is important to emphasize that continuous exposure of microorganisms to nanoparticles should be avoided, as a study on *E. coli* showed that bacteria can become resistant through 225 generations with constant exposure [65]. Unfortunately, silver nanoparticles have certain toxic effects in addition to their antibacterial properties. Many toxic effects of AgNPs have been demonstrated in in vitro studies. Research on the toxicity of AgNPs suggests that size, shape, chemical composition, solubility, surface activity, binding ability, and biological effects such as metabolism and excretion influence their toxicity [66]. The possibility of the AgNPs fixing in order to avoid their aggregation and to prevent their spontaneous release, especially in cases of medical application, seems to be a complementary advantage. 

### 2.6. Electrochemical Testing

Aqueous-based polymer electrolytes are commonly used as both separators and ionic conductors in solid-state devices. The advantage of polymer electrolytes over liquid electrolytes is their compact structure, which prevents liquid leakage and electrode displacement. This property is particularly important for flexible and free-standing supercapacitors, which have received considerable attention in recent years [67,68]. Most aqueous-based polymer electrolytes are prepared by blending polymer host materials with ionic conductors, water, and plasticizers [69]. Various fossil-based polymers: PET, aramid nonwovens and PP or PP/PP polyolefin membranes [70] have been investigated as separators for high power lithium ion batteries. In addition, poly(vinyl alcohol), which has excellent properties as a polymer host due to its high hydrophilicity and good film-forming ability is widely used as well as bio-based materials, among which cellulose is leading [71,72]. Recently, separators for high-performance supercapacitors (SCs) have been fabricated from keratin, a natural polymer with excellent wettability, which is an alternative sustainable material [73].

In this work, the polymer electrolytes prepared from 1-1_KS, 1-3_KS, and 1-5_KS hydrogels swollen in 0.5 M Na_2_SO_4_ were used as separators in supercapacitors with reduced graphene oxide/carbon nanotubes (rGO/CNT) as the active material. 

**Figure 13:**
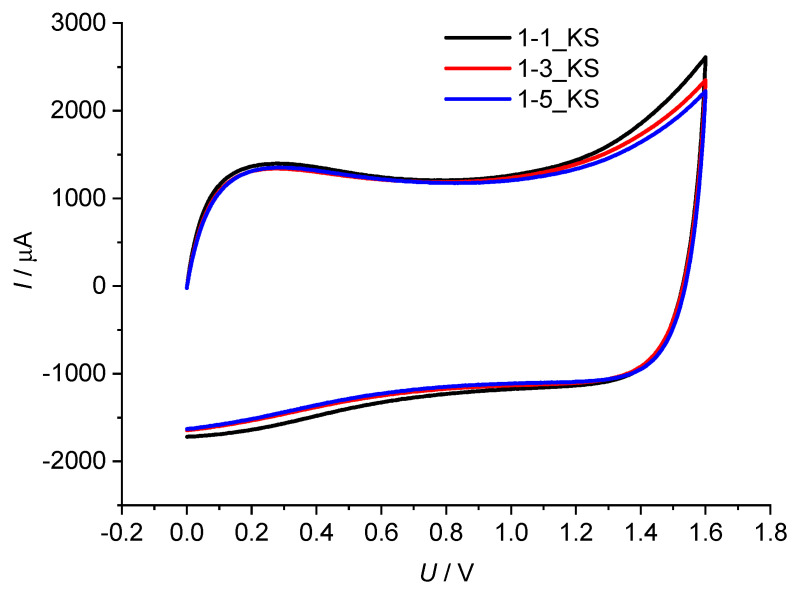
Responses obtained by cyclic voltammetry for rGO/CNT supercapacitor with three different hydrogels.

**Figure 14:**
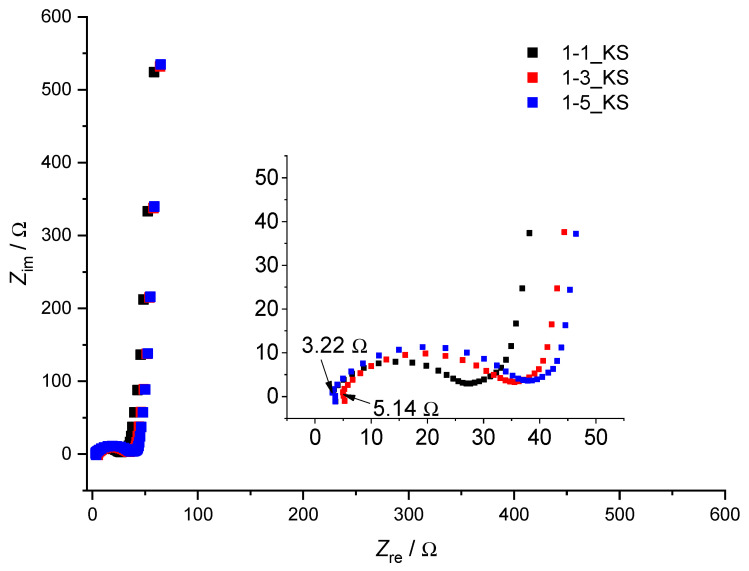
Electrochemical impedance spectroscopy responses obtained for rGO/CNT supercapacitor with three different hydrogels.

Figure 13 and Figure 14 show the CV responses of supercapacitors with different hydrogel electrolytes. The nearly constant current value registered during supercapacitor charging is related to the continuous electrochemical double layer charging by increasing the voltage [74,75,76]. In each case, a nearly rectangular response was obtained, indicating good capacitive behavior. There was no significant difference between the responses of the different hydrogels. The value of specific capacitance calculated according to Equation (2) was 25.86 F g^−1^ for 1-1_KS, 24.61 F g^−1^ for 1-3_KS and 24.34 F g^−1^ for 1-5_KS. The good capacitive behavior was confirmed by the EIS responses. In the high frequency region, a semicircle with two characteristic resistances was registered. The first one is related to the electrolyte resistance and the second one to the charge transfer resistance between the current collector and the active material. The total resistance represents the equivalent serial resistance (ESR) of the supercapacitor and determines the reversibility of the system and the charge/discharge rate of the supercapacitor. To improve the properties of the supercapacitor, it is of great importance to reduce the ESR. It is obvious that electrolyte resistance values for 1-1_KS and 1-5_KS were 3.2 Ω, while the resistance for 1-3_KS was 5.14 Ω. These values are very similar to those previously reported for supercapacitors assembled by using glass paper fibers wetted with 0.5 mol dm^−3^ Na_2_SO_4_ solution [74]. The low frequency response is related to the capacitance value. From the obtained results, it can be seen that the EIS response of each supercapacitor was similar, which is consistent with the results of CV. From these results, it can be concluded that the prepared hydrogel has good characteristics for supercapacitor application [77].

## 3. Conclusions

The methodology used enables the effective preparation of hydrogels based on cellulose and various ratios of PDMAEMA in the form of porous spheres or films. All prepared hydrogels displayed significant swelling ability. It reveals dependence on the composition of hydrogel, applied irradiation and pH while the achieved porosity of the material has a significant effect, as well. The materials showed steady swelling at acidic and base conditions over a period of two months. The introduction of silver particles and the application of gamma radiation have minimally increased the antibacterial activity of the prepared materials, which opens space for new research such as increasing the silver content or applying a higher dose of gamma radiation. The results from CV showed good capacitive behavior. Therefore prepared hydrogels seem to be promising materials for supercapacitors used as aqueous-based polymer electrolytes in separators in supercapacitors with rGO/CNT as an active material. 

## 4. Materials and Methods

### 4.1. Materials

Cellulose (*M*v = 25,100 g mol^−1^) was dissolved in the solvent *N,N*-dimethylacetamide (DMAc) (Fischer Chemical, analytical reagent grade) and lithium chloride (Fischer Chemical, laboratory reagent grade). Before dissolution in DMAc/LiCl solvent, cellulose was dried over phosphorus pentoxide (VWR, purity 99.5%) for 3 h at 90 °C under vacuum. DMAEMA monomer (polymerization grade, Aldrich) was passed through a column of activated basic aluminum oxide (Aldrich) and purged with high-purity nitrogen prior to use. The crosslinking agent MBA (Sigma-Aldrich, St. Louis, MI, USA, purity 99%) was used as received. The initiator of polymerization reaction tert-butylperoxy-2-ethylhexanoate (Trigonox 21, 70 wt% solution, Akzo Chemie, Amsterdam, The Netherlands) was used as received. Absolute ethanol (Gram-mol, p.a.) was used for freeze-extraction method as received. Silver nitrate (Alkaloid, p.a.) was used for the synthesis of silver particles. 

### 4.2. Method of Cellulose/Poly(dimethylaminoethyl methacrylate) Hydrogles (Cel-g-PDMAEMA) Synthesis

The samples of cellulose grafted with PDMAEMA were synthesized via free radical polymerization method using MBA as a cross-linker. The polymerization reaction was initiated by adding peroxide initiator. Synthesis was performed in two steps. Initially, cellulose was dispersed in DMAc in a round bottom flask and activated for 2 h at 120 °C. After two hours, the temperature was lowered to 100 °C and LiCl (6.6 wt%) was added to the flask and the mixture was stirred at 100 °C for another hour. The mixture was then cooled to room temperature and a clear cellulose solution (5 wt%) was obtained. Afterwards, the polymerization reaction of DMAEMA was carried out in a solution of cellulose in DMAc/LiCl. The obtained cellulose solution was weighed into a round bottom flask and heated to 90 °C. After 5 min, a solution of MBA and DMAEMA (50 wt%) in DMAc/LiCl was added to the flask. The weighted amount of initiator Trigonox 21 in DMAc/LiCl (1 wt% towards monomers) was added to the flask 5 min after the addition of monomers. The reaction was carried out for 3 h. After cooling, the synthesized polymers were precipitated in deionized water whereat the spheres were formed for the first phase of research. Hydrogel samples (spheres) were kept in deionized water for 5 days and the deionized water exchanged after 1 and 24 h, two and five days so as to remove all unreacted compounds. Afterwards spheres (samples) in the equilibrium swelling state were frozen and immersed in ethanol, dried in a freeze dryer, and used for further analysis. Weighed spheres (1 g) were placed in a beaker and cooled to −40 °C in a cryostat. The beaker containing the spheres was kept at −40 °C for 30 min and then cold ethanol (35 mL) was poured into the beaker. The beaker was placed in the freezer and the spheres were kept in ethanol at −18 °C for 48 h, with fresh ethanol added after 24 h. After 48 h, the ethanol was decanted and the spheres were dried in vacuum at 50 °C until constant weight. Molar ratio of reactants (monomer (DMAEMA), crosslinking agent (MBA) and cellulose (cel)) in Table 3.

#### 4.2.1. Gamma-Ray Irradiation of Hydrogel

The Cel-*g*-PDMAEMA hydrogel sample, which was in a rather viscous state, was subjected to a gamma-ray source, with gamma-ray absorbed doses of 10, 30 or 100 kGy [38]. The reaction mixture was placed in flasks, sealed and purged with N_2_ to achieve an oxygen-free environment, and then subjected to gamma irradiation. Irradiation was performed at the panoramic Co-60 gamma source of the Radiation Chemistry and Dosimetry Laboratory, Ruđer Bošković Institute. Gamma irradiation was used to further increase the conversion of the polymerization reaction, to graft PDMAEMA onto the cellulose and possibly achieve complete crosslinking of the hydrogel. The samples were irradiated for an appropriate duration with a dose rate of 19.8 kGy h^−1^. Dose mapping of the irradiation facility was performed experimentally using ionizing chambers and ECB dosimetric system, and by simulation calculations [78]. The resulting hydrogels were still in a liquid state, though more viscous. Initially, the resultant irradiated (10, 30 or 100 kGy) liquid material from Cel-*g*-PDMAEMA hydrogel was shaped into spheres, which were used for the study of swelling as well as structure characterization by FTIR. Based on the study of swelling of spheres the reaction mixture for preparation of hydrogel films was irradiated with the dose of 100 kGy. Molar ratio of reactants (monomer (DMAEMA), crosslinking agent (MBA) and cellulose (cel)) and irradiation dose for prepared samples are shown in Table 3.

#### 4.2.2. Preparation of Hydrogel Films

The liquid Cel-*g*-PDMAEMA hydrogels, originated from chemical synthesis as well as those irradiated (100 kGy), were casted on the glass plate and submersed into a deionized water bath. The deionized water was exchanged after 1 and 24 h, two and five days. The prepared hydrogel films were stored in a refrigerator. Samples were freeze-extracted using same procedure as for spheres. 

#### 4.2.3. Decorating Hydrogel Films with Silver Particles

Samples of 10 × 10 × 2 mm^3^ dimensions were cut from hydrogel films and used for testing antibacterial properties. Additionally, samples of the same dimensions were decorated with silver particles by immersing in 5 mL of silver nitrate solution (*c* = 10^−2^ mol dm^−3^). After 24 h, the samples were removed from the silver nitrate solution and rinsed with deionized water for 1 h to remove unreacted silver nitrate [79]. 

### 4.3. Structural Confirmation of Cel-g-PDMAEMA Hydrogels

#### 4.3.1. Fourier Transform Infrared Spectroscopy

Fourier transform infrared spectra (FTIR) were recorded using Perkin-Elmer Spectrum One equipped with ATR module at room temperature in frequency range 650–4000 cm^−1^. The FTIR spectra were processed using the OriginLab software.

#### 4.3.2. Scanning Electron Microscopy Analysis

Scanning electron microscopy (SEM) was used to examine the morphology of the synthesized Cel-*g*-PDMAEMA hydrogels. The lyophilized hydrogel samples were halved (spheres) or broken (films) and sputter coated by an alloy of gold and palladium (85%/15%) in the argon plasma to enhance their electrical conductivity. Metalized Cel-*g*-PDMAEMA hydrogel samples were scanned with a TESCAN VEGA 3 Scanning Electron Microscop, with a detector of secondary electrons.

#### 4.3.3. Swelling Study

A gravimetric method was used to measure the equilibrium swelling ratio of the Cel-*g*-PDMAEMA hydrogels. For swelling degree at least five spheres were swollen and the average result is presented. The swelling kinetic of freeze-extracted samples was carried out for 60 days. Dried samples were reswelled in deionized water pH value 5.5 as well as in acidic and basic solution, respectively, to determine the swelling degree. The equilibrium swelling weights were measured at RT for the hydrogel samples in a solution of an appropriate pH value (3.0, 5.5 and 10.0) after wiping excess water from the hydrogel surface with moistened filter paper. The masses of the samples were measured before starting and at defined intervals until equilibrium was reached, i.e., a constant sample mass was achieved. The swelling ratio, α, (and the equilibrium swelling ratio, α_e_,) were calculated according to Equation (1) [80]:

(1) $$\alpha =\frac{m_{t}-m_{0}}{m_{0}}\times 100\%$$

where *m*_0_ is mass of dry sample (xerogel) and *m*_t_ mass of swollen sample at the time *t*. 

### 4.4. X-ray Diffraction of Hydrogel Films Decorated with Silver Particles

Crystalline phase analysis was carried out using X-ray diffraction analysis (XRD) performed on Shimadzu XRD-6000 diffractometer with Cu K_α_ (1.5406 Å) radiation, operating at 40 kV, with a 2θ range of 30–90°, at a step size of 0.02°. The analyzed sample was ground into fine powder prior to XRD analysis.

### 4.5. Antibacterial Properties

The antimicrobial activity of the prepared films was tested against *Escherichia coli*, *Pseudomonas aeruginosa* and *Bacillus subtilis* by the agar disc diffusion test [81]. The specimens were placed (square shaped with an area of 1 × 1 cm^2^) on Mueller–Hinton sterilized agar (Mueller Hinton Agar, Biolife), and the agar plate was inoculated uniformly with 10^6^ CFU mL^−1^; lastly, the plates were incubated at 37 °C for 24 h. The volume of the added inoculum was 0.1 mL. The bacteria developed and enveloped the medium, except the resulting sterile area named “inhibition zone”, generated by the antimicrobial materials. After 24 h, the inhibition zone diameter was measured. Cellulose and 30 μL of sterile deionized water pipetted on to blank disc were used as a negative control, and Gentamicin (12 μg μL^−1^) as a positive control. All studies were performed in duplicate.

### 4.6. Electrochemical Testing

Two smooth nickel current collectors (1 × 1 cm^2^) spaced by polymer hydrogel sided with two rGO/CNT sheets were used in order to assemble a supercapacitor. The geometric area of the supercapacitor was 1 cm^2^. Three polymer hydrogels, 1-1_KS, 1-3_KS and 1-5_KS, were swollen in 0.5 M Na_2_SO_4_ for 30 min prior to testing. To determine the performance of the assembled supercapacitor, cyclic voltammetry (CV) and electrochemical impedance spectroscopy (EIS) were used. CV measurements were carried out at a scan rate of 50 mV s^−1^ in the voltage range between 0 and 1.2 V. EIS was performed in the frequency range between 10^5^ and 10^−3^ Hz using an AC voltage amplitude of ±5 mV at a DC voltage of 0 V. The measurements were carried out by using PalmSens potentiostat/galvanostat/impedance analyzer and PSTrace 5.8 Software. The resistance of polymer hydrogel electrolyte was calculated based on the EIS response of supercapacitor. Specific capacitance of the cells was calculated by integration of the cyclic voltammogram according to Equation (2) [74]:

(2) $$c_{s}=\frac{\int_{U_{1}}^{U_{2}}I\left(U\right)\cdot dU}{2mv\cdot \left(U_{2}-U_{1}\right)}$$

where *c_s_* is specific capacitance (F g^−1^), *I* current (A), *U* voltage (V), *U*_1_ starting voltage (V), *U*_2_ switching voltage (V), *m* mass of active material of one electrode (g), *ν* scan rate (V s^−1^). All tests were carried out under ambient conditions.

## Figures and Tables

**Table 1:** Intensity of characteristic bands and intensity ratio of carbonyl group and glycosidic bond in prepared hydrogels.

Sample	2940/ cm^−1^ (2925/ cm^−1^)	2850 / cm^−1^ (2821/ cm^−1^)	2770/ cm^−1^	1724/ cm^−1^	1156/ cm^−1^	1019/ cm^−1^	897/ cm^−1^	851/ cm^−1^	780/ cm^−1^	749/ cm^−1^	*I*_1724_/*I*_897_
1-1_KS	8.0	6.3	/	3.1	7.9	26.0	6.2	/	/	/	0.5
1-1_100	11.7	9.3	/	5.6	13.7	43.8	10.5	/	/	/	0.5
1-3_KS	11.3	8.7	5.7	16.8	19.5	34.6	3.8	1.9	1.6	2.1	4.5
1-3_100	6.6	4.9	5.5	17.9	19.7	22.7	1.7	2.5	2.5	3.3	10.5
1-5_KS	9.0	/	8.9	27.9	30.5	32.9	2.1	4.4	4.5	4.7	13.3
1-5_100	7.5	/	6.9	21.5	23.3	23.7	0.7	3.4	3.7	4.2	32.1

**Table 2:** Antimicrobial activity of tested samples using the agar disk diffusion method.

Sample	*d* (Inhibition Zone*–E. coli*)/cm	*d* (Inhibition Zone*–P. aeruginosa*)/cm	*d* (Inhibition Zone*–B. subtilis*)/cm
1-1 KS	0	0.05	0.1
1-1_KS + AgNO_3_	0.1	0.15	0.5
1-1_100 + AgNO_3_	0.2	0.25	0.7
1-3_KS	0	0	0
1-3_KS + AgNO_3_	0.7	0.4	0.5
1-3_100 + AgNO_3_	1	0.6	0.7
1-5_KS	0	0	0
1-5_KS + AgNO_3_	0.1	0.2	0.8
1-5_100 + AgNO_3_	0.6	0.4	1

**Table 3:** Molar ratio of reactants (monomer (DMAEMA), crosslinking agent (MBA) and cellulose (cel)) and irradiation dose for prepared samples.

Sample	*n*(cel)/*n*(DMAEMA)	*n*(DMAEMA)/*n*(MBA)	Irradiation Dose (kGy)	AgNO_3_
1-1_KS	1:1	15:1	0	-
*1-1_KS + AgNO_3_*			0	+
1-1_10			10	-
1-1_30			30	-
1-1_100			100	-
*1-1_100 + AgNO_3_*			100	+
1-3_KS	1:3	15:1	0	-
*1-3_KS + AgNO_3_*			0	+
1-3_10			10	-
1-3_30			30	-
1-3_100			100	-
*1-3_100 + AgNO_3_*			100	+
1-5_KS	1:5	15:1	0	-
*1-5_KS + AgNO_3_*			0	+
1-5_10			10	-
1-5_30			30	-
1-5_100			100	-
*1-5_100 + AgNO_3_*			100	+

## Supplementary Materials

The following supporting information can be downloaded at: https://www.mdpi.com/article/10.3390/gels8100636/s1, Figures S1–S3: Pore size distribution of prepared Cel-*g*-PDMAEMA samples and Figures S4–S6: Testing of antibacterial activity for prepared hydrogel samples.
