# Supplementary material for: Cellulose-g-poly(2-(dimethylamino)ethylmethacrylate) Hydrogels: Synthesis, Characterization, Antibacterial Testing and Polymer Electrolyte Application
Source: Gels. 2022 Oct 7;8(10):636. doi: 10.3390/gels8100636 (PMC9601901; doi:10.3390/gels8100636)
Supplement: Supplementary file 1 [file gels-08-00636-s001.zip › gels-1892031-supplementary.pdf]

gels

MDPI

Article

Cellulose-*g*-poly(2-(dimethylamino)ethylmethacrylate) Hydrogels: Synthesis, Characterization, Antibacterial Testing and Polymer Electrolyte Application

Roko Blažic, Dajana Kučić Grgić, Marijana Kraljić Roković and Elvira Vidović *

Supplement Materials

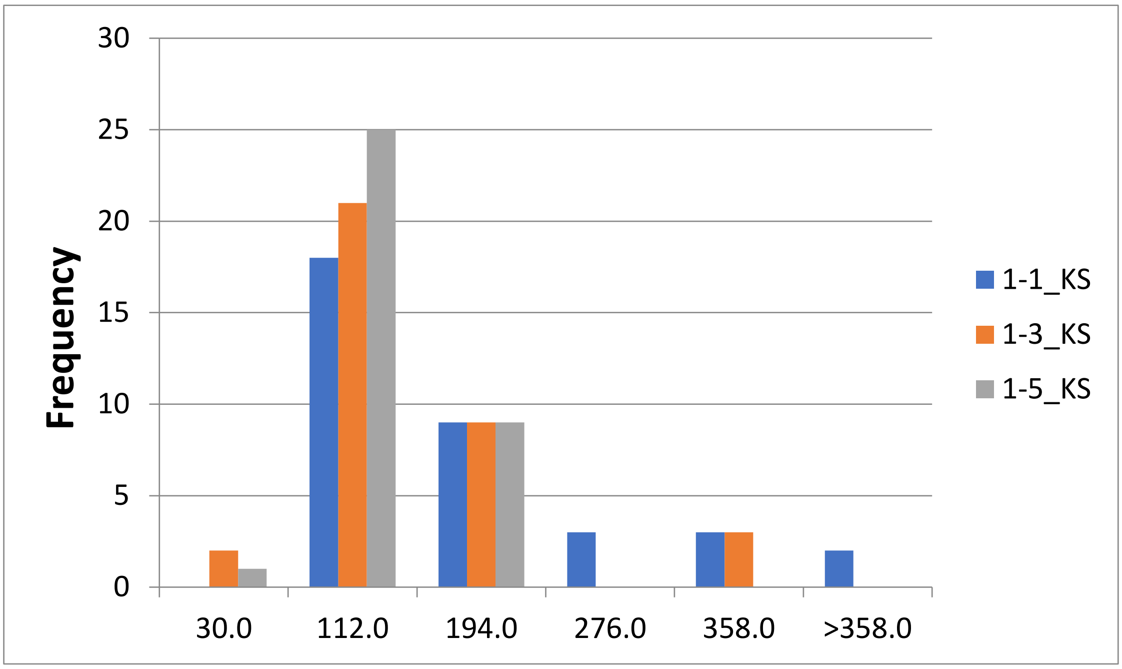

Figure S1. Pore size distribution of samples prepared by chemical synthesis and shaped into spheres.

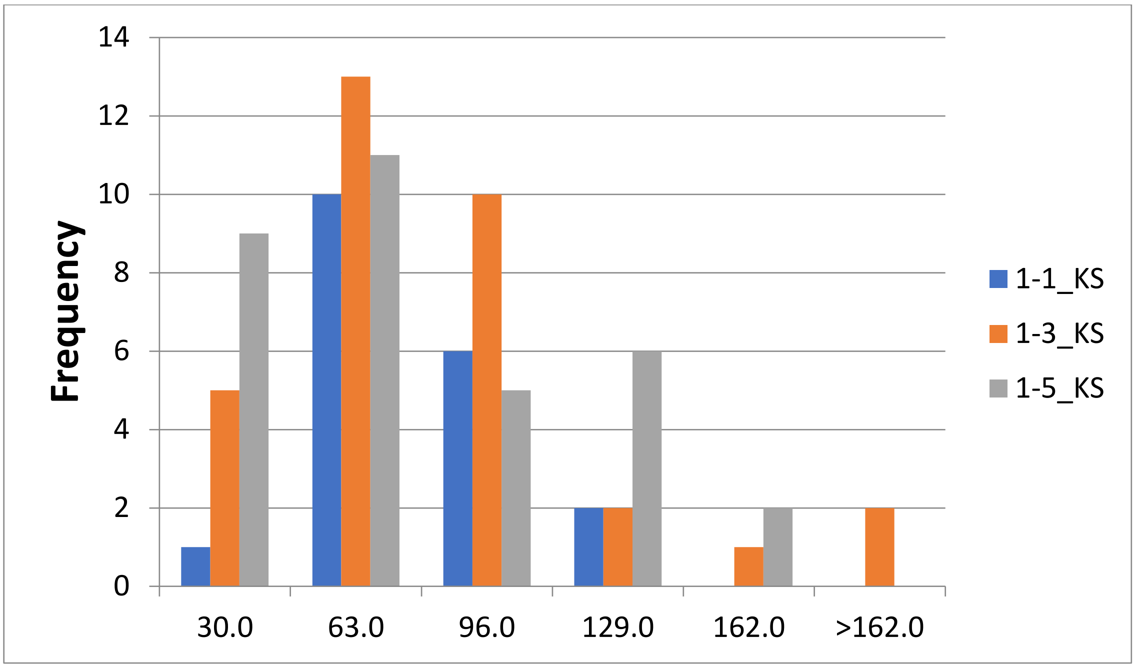

Figure S2. Pore size distribution of samples prepared by chemical synthesis and shaped hydrogel films.

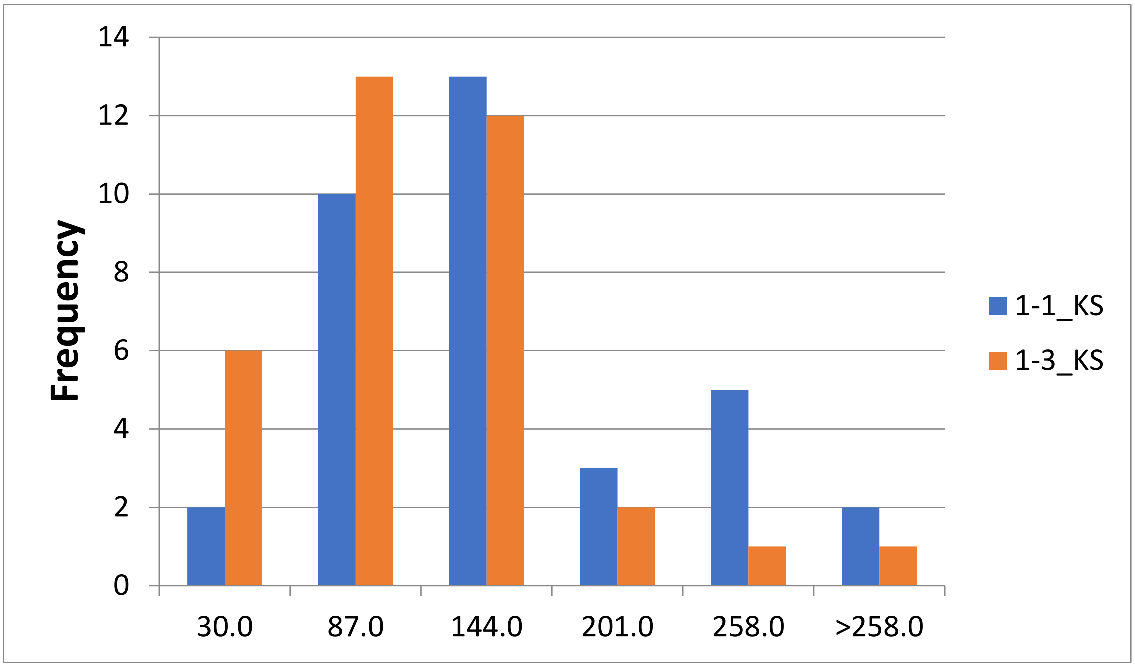

Figure S3. Pore size distribution of spherical samples after 60 days of swelling in deionized water.

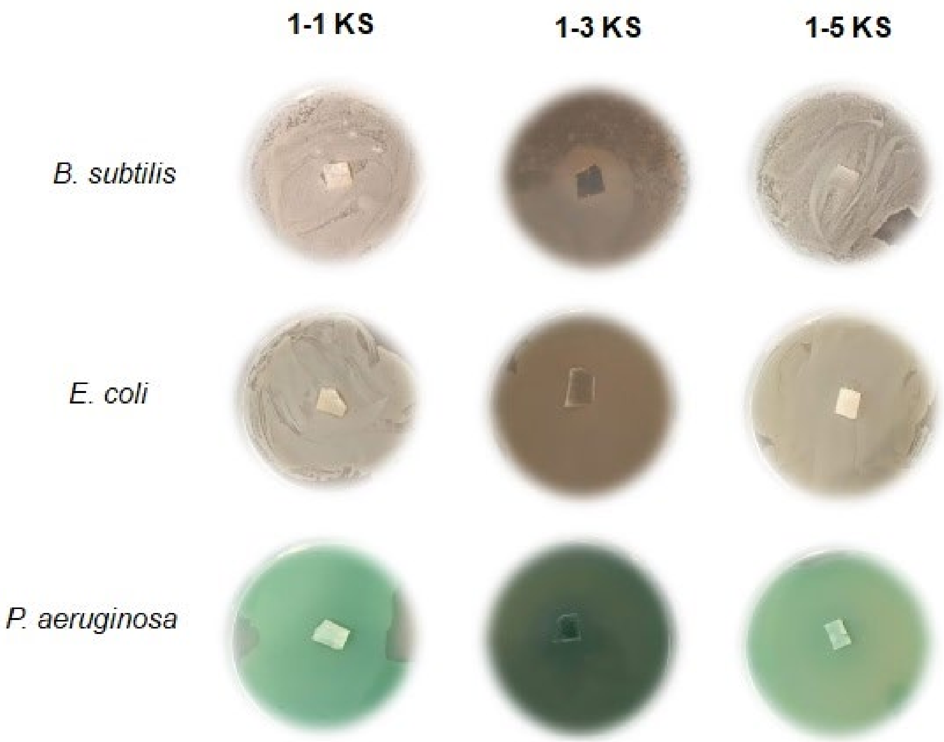

Figure S4. Antibacterial activity of chemically synthesized hydrogels.

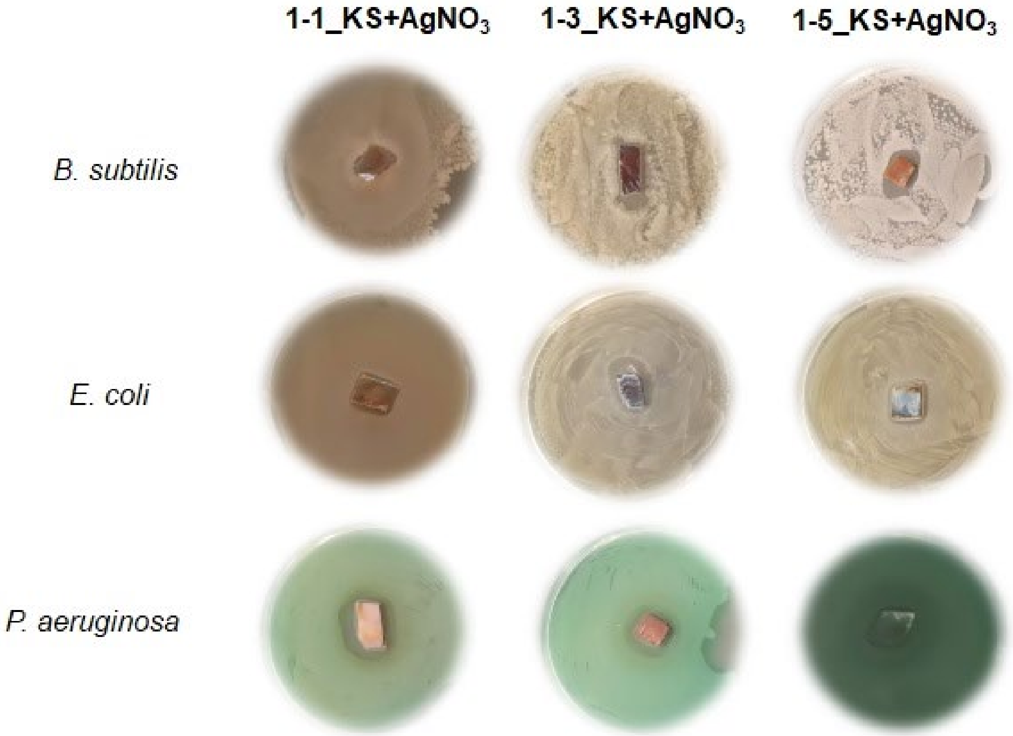

Figure S5. Antibacterial activity of chemically synthesized hydrogels additionally decorated with silver.

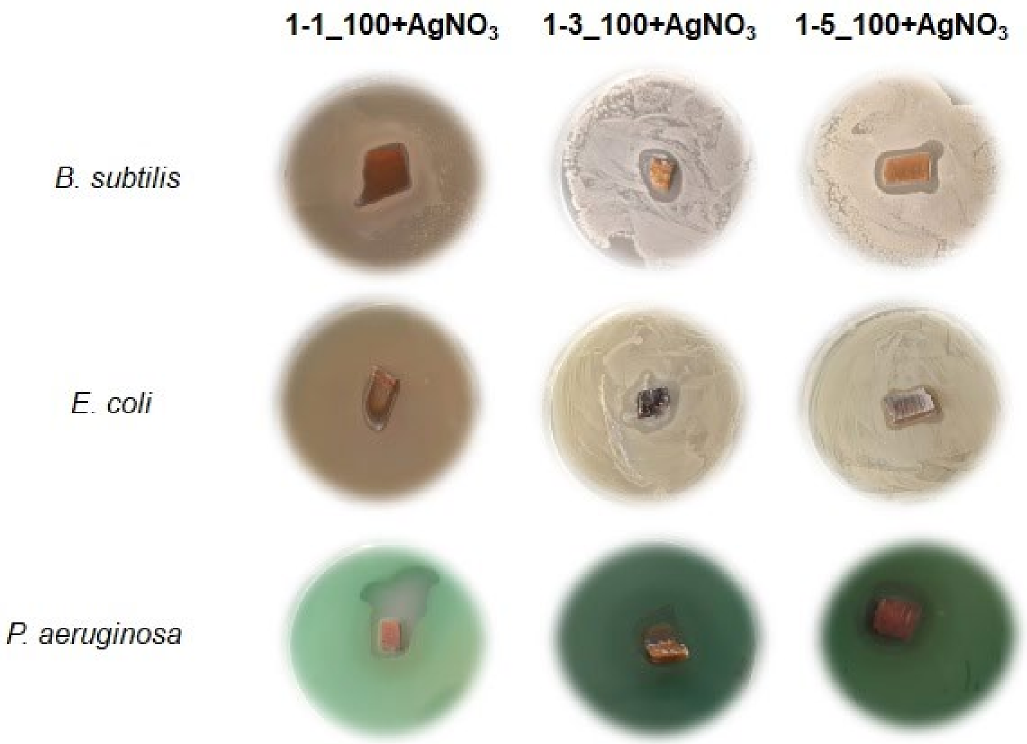

Figure S6. Antibacterial activity of chemically synthesized hydrogels additionally irradiated and decorated with silver.
